# Effect of Spraying Power on Oxidation Resistance of MoSi_2_-ZrB_2_ Coating for Nb-Si Based Alloy Prepared by Atmospheric Plasma

**DOI:** 10.3390/ma13225060

**Published:** 2020-11-10

**Authors:** Guanqun Zhuo, Linfen Su, Kaiyong Jiang, Jianyong Yang

**Affiliations:** 1 College of Mechanical Engineering and Automation, Huaqiao University, Xiamen 361021, China; zhuoguanqun@hqu.edu.cn (G.Z.); kyjiang@hqu.edu.cn (K.J.); yangjianyong@hqu.edu.cn (J.Y.); 2 Fujian Key Laboratory of Special Energy Manufacturing, Huaqiao University, Xiamen 361021, China

**Keywords:** Nb-Si based alloy, MoSi_2_-ZrB_2_ coating, atmospheric plasma, oxidation

## Abstract

The MoSi_2_-ZrB_2_ coatings were prepared on Nb-Si based alloy by atmospheric plasma spraying with the spraying power 40, 43 and 45 kW. The effect of spraying power on the microstructure and oxidation resistance of MoSi_2_-ZrB_2_ coating at 1250 °C were studied. The results showed that the main constituent phases of coatings were MoSi_2_ at all spraying power. The coating became more compact as the spraying power increased. The coating prepared at 45 kW was dense and uniform, which exhibited the best oxidation resistance due to the formation of a dense and uniform glass layer consisting of SiO_2_ and ZrSiO_4_.

## 1. Introduction

Nb-Si based alloys are considered to be one of the most promising high-temperature structural material, owing to the high melting points (1750 °C), medium density and excellent high-temperature strength [1,2,3,4,5,6,7]. However, the high-temperature oxidation resistance of Nb-Si based alloys are poor, which limits their application [8,9,10]. Adding elements such as Cr, B, Ta, Ti, Si in Nb-Si based alloys or preparing coatings on the Nb-Si based alloys can improve the oxidation resistance; however, alloying would compromise mechanical properties of alloys [11,12]. Therefore, more attention is paid to preparing coatings on Nb-Si based alloys.

MoSi_2_ is a promising coating material for Nb-Si based alloy [13,14,15,16]. It can produce SiO_2_ oxide film with excellent oxidation resistance at high temperatures. Besides, its thermal expansion coefficient (8.1 × 10^−6^ °C^−1^) is close to that of Nb-Si based alloys (8.4 × 10^−6^ °C^−1^) [17]. However, MoSi_2_ would suffer pest oxidation at 400–600 °C. The addition of B can effectively avoid this disadvantage [18]. The formation of borosilicate glass at high temperatures can effectively protect MoSi_2_ against pest oxidation at 400–600 °C [18]. Furthermore, borosilicate glass coating has a better self-repair ability than SiO_2_ due to its lower viscosity [19,20,21]. Fu et al. prepared B_2_O_3_ modified SiC-MoSi_2_ coating on C/C composites by a two-step pack cementation [22]. The coating could protect C/C composites from oxidation at 1500 °C in air for more than 242 h. Pang et al. prepared a Mo-Si-B coating on Nb-Si-based alloys by spraying Mo first and then co-deposition of Si and B [23]. The mass gain was 0.92 mg/cm^2^ after oxidation at 1250 °C for 100 h. However, stresses caused by CTE mismatch between the MoSi2 coating and silica can produce cracks in the oxide film if they exceed the strength of the SiO_2_.

Atmospheric plasma spraying has attracted widespread attention due to the advantages such as high spraying temperature, high deposition efficiency and precise control of the composition and thickness of the coating [24,25]. Le et al. directly deposited the oxidation-resistant coating MoSi_2_ on Nb alloy substrate by supersonic air plasma spraying with pure agglomerated MoSi_2_ powder [25]. After oxidation at 1500 °C in air for 43 h, it showed excellent oxidation resistance with mass loss of 5.31 mg cm^2^. Some of the literature has mentioned that the addition of Zr to Mo-Si-B coating could effectively improve the mechanical properties of MoSi_2_ at a high temperature. Furthermore, the ZrSiO_4_ produced by the reaction of dispersive ZrO_2_ and SiO_2_ could minimize the CTE difference between silica and MoSi_2_, as well as the consumption of SiO_2_ at a high temperature [26,27,28]. In this study, the MoSi_2_-ZrB_2_ coatings were prepared on Nb-Si based alloy by the atmospheric plasma spraying technology. The effects of spraying power on coating structure and the oxidation mechanism of MoSi_2_-ZrB_2_ coating were investigated.

## 2. Materials and Methods

### 2.1. Preparation of MoSi_2_-ZrB_2_ Coating

Substrates (Nb-15Si-24Ti-13Cr-2Al-2Hf (at.%) were fabricated by non-consumable arc-melting. The ingots were re-melted and inverted at least four times to guarantee the uniformity of the composition. Samples with a size of 10 × 10 × 8 mm^3^ were cut from the ingots. All surfaces were mechanically ground on wet SiC paper to 800 grit, then cleaned ultrasonically with ethanol and dried at about 80 °C for 1 h. Commercially available MoSi_2_ with 95 wt.%, ZrB_2_ with 5 wt.% powders were selected as raw materials with the purity of 99.9 wt.% and the particle size between 45 and 65 μm. The powders were ground in a planetary ball mill for 2 h, to ensure their uniformity. The MoSi_2_-ZrB_2_ coatings were prepared by atmospheric plasma spraying, at the power of 40, 43 and 45 kW, respectively. The samples were designated as mz40, mz43 and mz45, according to the spraying power. The spraying distance was set as 100 mm. Argon was used as primary gas and carrier gas, and hydrogen was used as secondary gas. The detailed parameters are listed in Table 1.

### 2.2. Isothermal Oxidation

An isothermal oxidation test was carried out in an open tube furnace, in air, at 1250 °C. Each sample was placed in a separate alumina crucible. Samples were taken from the furnace at intervals of 10, 20, 40 and 60 h, and weighed with a crucible, using a precision analytical balance (model CPA225D, Sartorius, Göttingen, Germany) with an accuracy of 0.00001 g.

### 2.3. Coating Characterization

Phase composition of the coating and oxidation specimens were analyzed by X-ray diffraction (XRD, CuKα-radiation, X’Pert Pro, Panalytical, Almelo, Holland) with Cu radiation. Morphology details and elemental distribution characteristics of the coated specimens were investigated by scanning electron microscope combined with energy dispersive spectroscopy (EDS) (Sigma 500, Zeiss, Oberkochen, Germany).

## 3. Results

### 3.1. Microstructure of MoSi_2_-ZrB_2_ Coating

**Figure 1:**
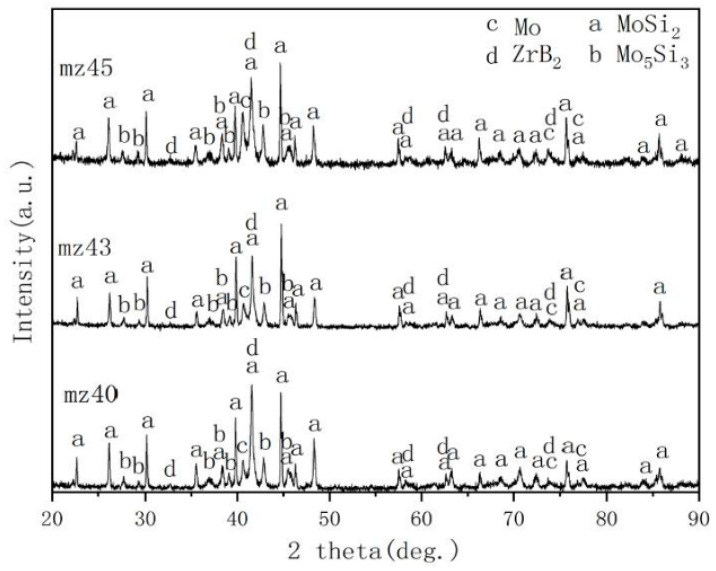
XRD patterns of MoSi_2_-ZrB_2_ coatings.

Figure 1 shows the XRD patterns of the surface of as-prepared MoSi_2_-ZrB_2_ coatings. It could be seen that the constituent phases of coating at different spraying powers are MoSi_2_, Mo_5_Si_3_, Mo and ZrB_2_. In the process of plasma spraying, the temperature of the plasma arc was about 10,000 °C [28], which is much higher than the oxidation temperature of MoSi_2_. Therefore, the raw materials are oxidized to form Mo_5_Si_3_, SiO_2_ and Mo according to the Equations (1) and (2) [29,30,31,32]. SiO_2_ is an amorphous phase, and its amount is relatively small; therefore, no SiO_2_ phase is detected in XRD patterns.

5MoSi_2_ + 7O_2_ → Mo_5_Si_3_ + 7SiO_2_ (1)

MoSi_2_ + 2O_2_ → Mo + 2SiO_2_ (2)

**Figure 2:**
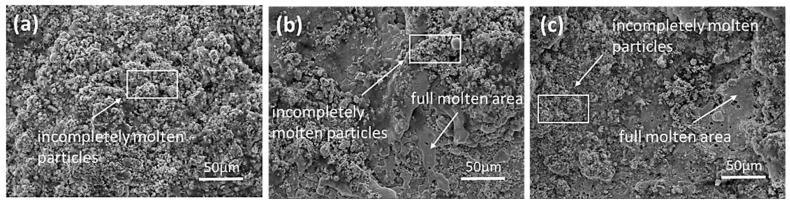
Surface morphology of MoSi_2_-ZrB_2_ coatings: (**a**) mz40, (**b**) mz43 and (**c**) mz45.

Figure 2 shows the surface morphology of MoSi_2_-ZrB_2_ coatings. The surfaces of all spraying samples are rough. In addition, the molten zone is interwoven with the incompletely molten particles, which is a typical structure feature of plasma sprayed coatings. In the process of coating preparation, high-speed particles are heated by plasma flame, and then the molten particles impinge on the substrate to form a flat structure. The temperature of the plasma arc elevates as the spraying power increases. Therefore, the full molten area of the mz43 sample is larger than that of the mz40 sample, leading to a much smoother surface of the mz43 sample. As for the mz45 sample, the completely molten area of the mz45 sample is the largest (as shown in Figure 2c). Therefore, the mz45 sample shows a more compact surface as compared with that of the mz40 and mz43 samples.

**Figure 3:**
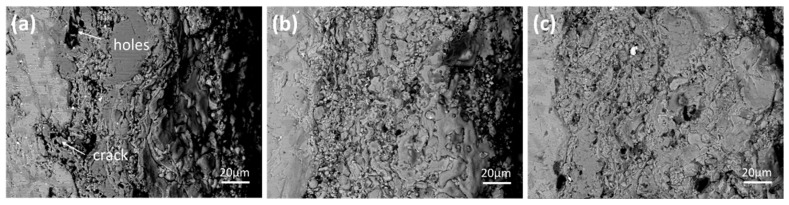
Cross-section morphology of MoSi_2_-ZrB_2_ coatings: (**a**) mz40, (**b**) mz43 and (**c**) mz45.

**Figure 4:**
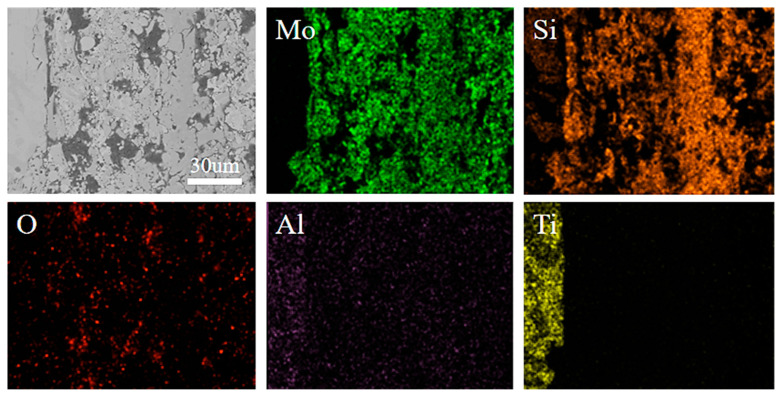
Elements mapping for the MoSi_2_-ZrB_2_ coating of the mz45 sample.

Figure 3 shows cross-sectional morphologies of MoSi_2_-ZrB_2_ coatings. The mean thickness of the coatings of the mz40, mz43 and mz45 samples are about 122, 138 and 158 μm, respectively. As shown in Figure 3, the interface between the coating and the substrate becomes denser and more uniform as the power increases. For the mz45 sample, the interface is more compact, indicating that the mz45 sample has a better combination of the coating and substrate as compared to that of the mz40 and mz43 samples. As shown in Table 2, the main constituent phase of the mz45 sample is confirmed to be MoSi_2_. The elements mapping, as shown in Figure 4, reveals that the coating mainly consists of Mo, Si and O. The existence of O element may be induced from the spraying in oxygen atmosphere. Furthermore, the Vickers hardness of the coating prepared by 40, 43 and 45 Kw are measured to be 850, 924 and 979, respectively. This may be due to the better combination of the substrate and coating as the increase of the spraying power.

### 3.2. High Temperature Oxidation Resistance

**Figure 5:**
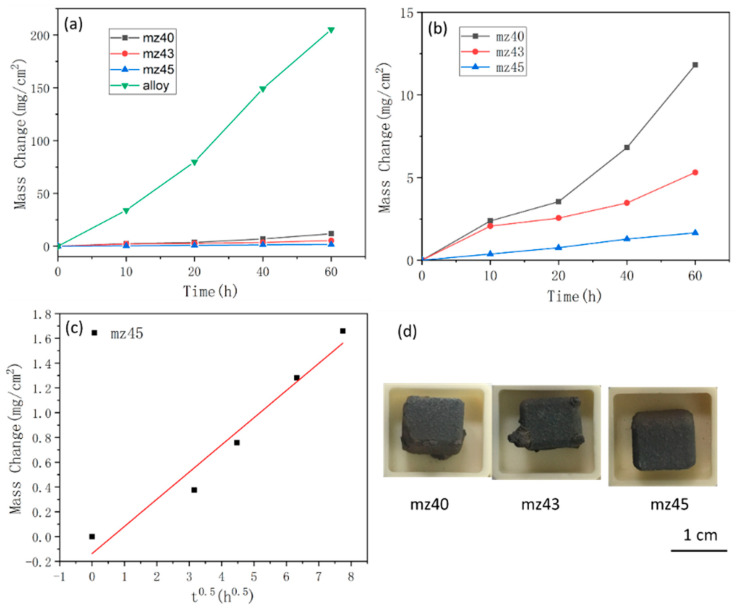
(**a**) Oxidation weight gain curve of Nb-Si based alloy and coatings, (**b**) oxidation weight gain curve of coatings, (**c**) representation of the weight gain versus the square root of time for mz45 oxidized in air, and (**d**) the photograph of oxidized samples.

Figure 5a shows the weight gain per unit area as a function of the exposure time at 1250 °C. The Nb-Si based alloy suffers serious oxidation with the mass gain of 205.24 mg cm^−2^ after oxidation at 1250 °C for 60 h and follows a linear oxidation behavior. The mass gains of the mz40, mz43 and mz45 samples were 11.81, 5.32 and 1.66 mg/cm^2^, respectively. Therefore, MoSi_2_-ZrB_2_ coatings could effectively improve the oxidation resistance of Nb-Si based alloy. As shown in Figure 5b, the mz40 and mz43 samples follow linear oxidation behavior, and the linear kinetic constants (g^2^/cm^4^s) of the mz40 and mz43 sample are calculated to be 1.89 × 10^−5^ and 7.8 × 10^−6^, respectively, according to Equation (3) [4], where Δm is the weight change of the sample, *A* is the surface area and t is the exposure time. During oxidation, the edges of the coating are the place where stress is easily concentrated, leading to the failure of the coating. As shown in Figure 5d, the coating edges of the mz40 and mz43 samples have peeled off after oxidation, while the coating edge of mz45 sample is compact. The mz45 sample shows excellent high temperature oxidation resistance and conforms to the parabolic oxidation behavior. The parabolic kinetic constant (g^2^/cm^4^s) of the mz45 sample is calculated to be 1.27 × 10^−11^ according to Equation (4) [17], where Δm is the weight change of the sample, and *A* is the surface area and t is the exposure time.

(3) $$\frac{\Delta m}{A}=k_{l}t$$

(4) $${\left(\frac{\Delta m}{A}\right)}^{2}=k_{p}t$$

**Figure 6:**
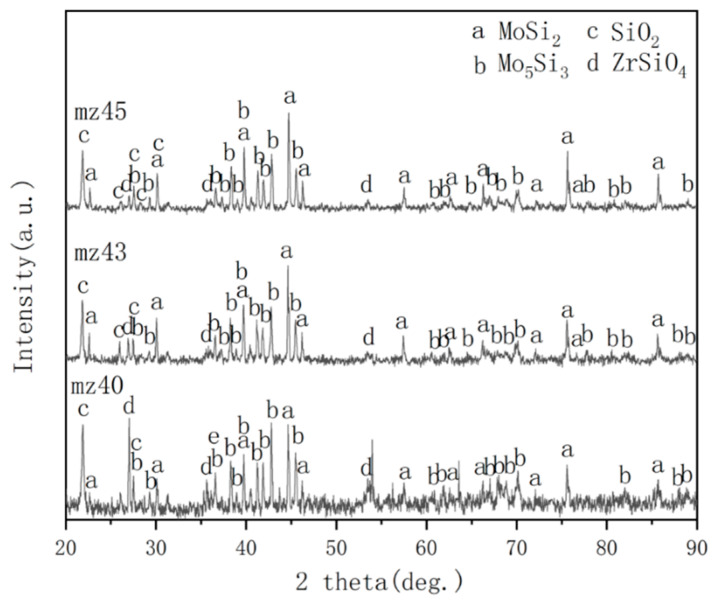
XRD patterns of MoSi_2_-ZrB_2_ coatings after oxidation at 1250 °C for 60 h.

**Figure 7:**
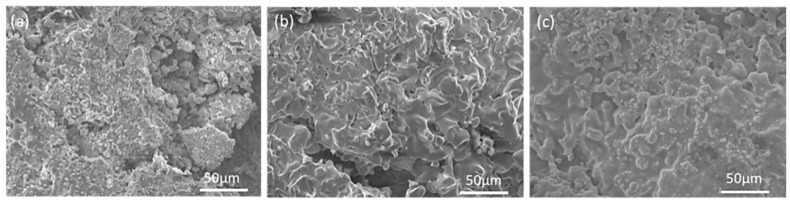
Surface morphology of MoSi_2_-ZrB_2_ coatings after oxidation in air at 1250 °C for 60 h: (**a**) mz40, (**b**) mz43 and (**c**) mz45.

Figure 6 shows the XRD patterns of MoSi_2_-ZrB_2_ coatings after oxidation at 1250 °C for 60 h. As shown in Figure 6, the oxidized MoSi_2_-ZrB_2_ coatings mainly consist of MoSi_2_, Mo_5_Si_3_, SiO_2_ and ZrSiO_4_. Figure 7 demonstrates the surface morphologies of MoSi_2_-ZrB_2_ coatings after oxidation at 1250 °C for 60 h. The surface of the mz40 sample is loose and undulate, as shown in Figure 7a. It can be observed from Figure 7b that the surface of mz43 sample is much denser. As shown in Figure 7c, the mz45 sample displays a uniform, dense and integrated surface.

**Figure 8:**
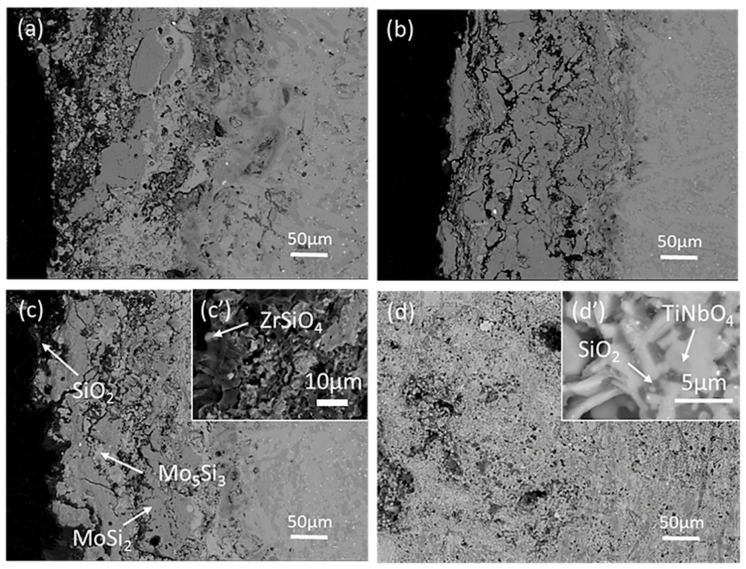
Cross-section morphology of MoSi_2_-ZrB_2_ coatings after oxidation in air at 1250 °C for 60 h: (**a**) mz40, (**b**) mz43, (**c**) mz45 and (**d**) the failure edge of the mz40 sample.

Figure 8 shows the cross-sectional morphologies of MoSi_2_-ZrB_2_ coatings after oxidation at 1250 °C for 60 h. According to the XRD results and EDS analysis (as listed in Table 3), the coating consists of the black SiO_2_, the black-gray MoSi_2_, the gray-white Mo_5_Si_3_ and the white ZrSiO_4_. Moreover, some cracks and holes are observed in all coatings, and they are filled with black SiO_2_ phase. As shown in Figure 5d, the coating edges of the mz40 and mz43 samples are peeling off during oxidation. Therefore, the edge of the substrate (Nb-Si based alloy) exposes to the oxygen environment, leading to the worse oxidation performance of these two samples. Figure 8d shows the microstructure of the failure edge of the mz40 sample after oxidation. The oxides of the edge of mz40 sample are confirmed to be TiNbO_4_ and SiO_2_ according to EDS analysis, which is the typical oxides of Nb-Si based alloy at high temperature [17].

## 4. Discussion

### 4.1. Effect of Spraying Powder on the Microstructure of Coatings

As shown in Figure 2, the surface of the mz45 sample is more compact due to the completely molten area of the mz45 sample is the largest. In the process of spraying, the temperature of the plasma arc elevates as the spraying power increases. The plasma arc of 40 kW possesses a lower temperature. Therefore, the surface of the mz40 sample is rough consisting of many incompletely molten particles and holes, which is mainly due to the accumulation of incompletely molten particles and the escape of gas by-products during the spraying process. In addition, the incompletely molten powders are difficult to adhere to the substrate surface, which reduces the spraying efficiency, resulting in the thinnest thickness of the coating.

When the spraying power increases to 43 kW, the increasing temperature increases the melting ratio of spraying powder. These spraying powders can be riveted together with the substrate when they hit the surface of the substrate. Thus, the completely molten area increases in this sample. However, a few holes still exist at the interface between the coating and the substrate, suggesting that the spraying powders cannot be fully spread at 43 kW. When the power increases to 45 kW, completely molten particles can be uniformly distributed on the surface of the substrate, and the thickness of the coating increases. In addition, no obvious cracks and holes were observed at the interface between the coating and the substrate, indicating the good bonding of the mz45 sample. It could be concluded that the higher spraying power could produce a much more compact coating.

### 4.2. Oxidation Mechanism of MoSi_2_-ZrB_2_ Coatings

As shown in Figure 8, the oxidized MoSi_2_-ZrB_2_ coatings mainly consist of MoSi_2_, Mo_5_Si_3_, SiO_2_ and ZrSiO_4_. The excellent oxidation resistance of the mz45 sample is due to the formation of dense SiO_2_ glass layer on the surface, leading to a lower diffusion rate of oxygen. The existence of Mo_5_Si_3_ phase is due to the oxidation of MoSi_2_ phase according to the oxidation reaction (Equation (1)) [30,31]; the formation of SiO_2_ phase is due to the oxidation of silicides, such as MoSi_2_ and Mo_5_Si_3_ according to Equations (5) and (6) [30,31,32,33]; ZrB_2_ are oxidized to form ZrO_2_ and B_2_O_3_ according to Equation (7). The ZrSiO_4_ phase is the result of reaction between the SiO_2_ and ZrO_2_ according to Equation (8). Owing to the volatilization of MoO_3_ and B_2_O_3_ at high temperature, the MoO_3_ phase and B_2_O_3_ phase cannot be observed in the coating. Although the oxidation protective phase SiO_2_ is formed in the mz40 and mz43 samples, these two samples exhibit worse oxidation due to the bad combination between coating and substrate at lower spraying power.

MoSi_2_ + 7O_2_ → 2MoO_3_ + 4SiO_2_ (5)

2Mo_5_Si_3_ + 21O_2_→10MoO_3_ + 6SiO_2_ (6)

ZrB_2_ + 5O_2_→2ZrO_2_ + 2B_2_O_3_ (7)

ZrO_2_ + SiO_2_ → ZrSiO_4_ (8)

Moreover, it can be found that ZrSiO_4_ distributed in the coating, as shown in Figure 8c. Dissolving a certain amount of zirconium oxide in amorphous silica scale could enhance its oxidation resistance [27]. Zr-based oxides have higher melting temperature, of which ZrO_2_ is 2715 °C, ZrSiO_4_ is 2550 °C, and pure SiO_2_ is 1650 °C [34,35,36]. Therefore, the dispersion of ZrSiO_4_ in SiO_2_ glass could increase the melting temperature of the silica. Furthermore, the CTEs of ZrO_2_ (10.5 × 10^−6^ °C^−1^) and ZrSiO_4_ (4.9 × 10^−6^ °C^−1^) are larger than that of SiO_2_ (0.55 × 10^−6^ °C^−1^) [37,38,39,40]. Therefore, the formation of ZrSiO_4_ could increase the CTE of silica. As a result, the difference of CTE between silica and MoSi_2_ could minimize, reducing the internal stress of the coating.

**Figure 9:**
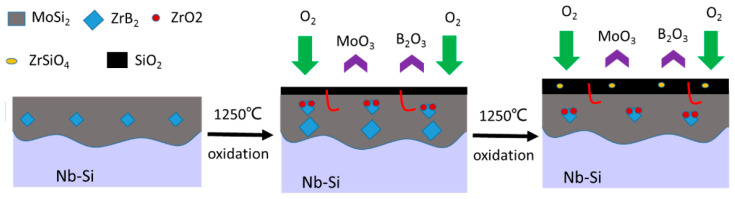
Oxidation process of MoSi_2_-ZrB_2_ coating at 1250 °C in air.

In order to explain the oxidation mechanism of MoSi_2_-ZrB_2_ coating, the oxidation process is shown in Figure 9, which is similar to that of MoSi_2_-based composite coating on Nb alloy at 1500 °C [25]. MoSi_2_ and ZrB_2_ are oxidized to form SiO_2_ and ZrO_2_, respectively. After that, the silica glass covers the surface of the coating and heals the cracks and holes. As oxidation continued, the ZrSiO_4_ is produced by the reaction of dispersive ZrO_2_ and SiO_2_, which could minimize the CTE difference between silica and MoSi_2_.

## 5. Conclusions

The effect of different spraying power on the microstructure and oxidation resistance of the MoSi_2_-ZrB_2_ coatings was investigated.

1. The microstructure of MoSi_2_-ZrB_2_ coatings prepared by atmospheric plasma spraying mainly consisted of MoSi_2_. The higher spraying power could produce a much more compact coating.
2. The MoSi_2_-ZrB_2_ coating prepared under 45 Kw showed the best oxidation resistance with the mass gain of 1.66 mg cm^-2^ after oxidation at 1250 °C for 60 h. However, the MoSi_2_-ZrB_2_ coatings prepared under 40 and 43 Kw showed worse oxidation resistance, due to the bad combination between coating and substrate at the lower spraying power.
3. The excellent anti-oxidation protection of mz45 sample was mainly due to the formation of a silica glass layer, leading to a low diffusion rate of oxygen.

## Figures and Tables

**Table 1:** Detailed parameters of the sprayed MoSi_2_-ZrB_2_ coating.

Content	Parameters
Spraying power (kW)	40–45
Primary gas Ar (L/min)	58
Carrier gas Ar (L/min)	10
Second gas H_2_ (L/min)	9
Powder feed rate (g/min)	17
Spraying distance (mm)	100
Nozzle diameter (mm)	5.5

**Table 2:** The EDS results of constituent phase of mz45 sample (at.%).

Elements	Mo	Si	O	B
MoSi_2_	34.29	65.71	0	0

**Table 3:** The EDS results of constituent phase of the oxidized mz45 sample (at.%).

Element	Mo	Si	Zr	B	Nb	O
SiO_2_	0	38.26	0	0	0	61.74
MoSi_2_	32.99	67.01	0	0	0	0
Mo_5_Si_3_	52.02	35.4	0	2.93	3.08	6.57
ZrSiO_4_	0	15.01	14.91	0	0	70.08

## References

[1] Bewlay B.P., Jackson M.R., Subramanian P.R., Zhao J.-C. (2003). A review of very-high-temperature Nb-silicide-based composites. Met. Mater. Trans. A.

[2] Tang Y., Guo X. (2016). High temperature deformation behavior of an optimized Nb–Si based ultrahigh temperature alloy. Scr. Mater..

[3] Guan K., Jia L., Kong B., Yuan S., Zhang H. (2016). Study of the fracture mechanism of NbSS/Nb_5_Si_3_ in situ composite: Based on a mechanical characterization of interfacial strength. Mater. Sci. Eng. A.

[4] Geng J., Tsakiropoulos P., Shao G. (2006). The effects of Ti and Mo additions on the microstructure of Nb-silicide based in situ composites. Intermetalic.

[5] Zhang S.-N., Guo Y., Kong B., Zhang F.-X., Zhang H. (2017). High-temperature oxidation behavior of Nb-Si-based alloy with separate vanadium, tantalum, tungsten and zirconium addition. Rare Met..

[6] Bewlay B.P., Jackson M.R., Zhao J.-C., Subramanian P.R., Mendiratta M.G., Lewandowski J.J. (2003). Ultrahigh-Temperature Nb-Silicide-Based Composites. MRS Bull..

[7] Bewlay B.P., Jackson M.R., Lipsitt H.A. (1996). The balance of mechanical and environmental properties of a multielement niobium-niobium silicide-basedIn Situ composite. Met. Mater. Trans. A.

[8] Wang L., Jia L., Cui R., Zheng L., Zhang H. (2012). Microstructure, Mechanical Properties and Oxidation Resistance of Nb-22Ti-14Si-2Hf-2Al-xCr Alloys. Chin. J. Aeronaut..

[9] Li X., Chen H., Sha J., Zhang H. (2010). The effects of melting technologies on the microstructures and properties of Nb–16Si–22Ti–2Al–2Hf–17Cr alloy. Mater. Sci. Eng. A.

[10] Yao D., Cai R., Zhou C., Sha J., Jiang H. (2009). Experimental study and modeling of high temperature oxidation of Nb-base in situ composites. Corros. Sci..

[11] Kang Y., Qu S., Song J., Huang Q., Han Y. (2012). Microstructure and mechanical properties of Nb–Ti–Si–Al–Hf–xCr–yV multi-element in situ composite. Mater. Sci. Eng. A.

[12] Geng J., Tsakiropoulos P. (2007). A study of the microstructures and oxidation of Nb–Si–Cr–Al–Mo in situ composites alloyed with Ti, Hf and Sn. Intermetalic.

[13] Xiao L.-R., Cai Z.-G., Yi D.-Q., Ying L., Liu H.-Q., Huang D.-Y. (2006). Morphology, structure and formation mechanism of silicide coating by pack cementation process. Trans. Nonferrous Met. Soc. China.

[14] Yan J., Wang Y., Liu L., Wang Y. (2014). Oxidation and interdiffusion behavior of Niobium substrate coated MoSi_2_ coating prepared by spark plasma sintering. Appl. Surf. Sci..

[15] Hidouci A., Pelletier J. (1998). Microstructure and mechanical properties of MoSi_2_ coatings produced by laser processing. Mater. Sci. Eng. A.

[16] Sun S., Li X., Wang H., Jiang Y., Yi D. (2015). Prediction on anisotropic elasticity, sound velocity, and thermodynamic properties of MoSi_2_ under pressure. J. Alloy. Compd..

[17] Su L., Jia L., Weng J., Hong Z., Zhou C., Zhang H. (2014). Improvement in the oxidation resistance of Nb–Ti–Si–Cr–Al–Hf alloys containing alloyed Ge and B. Corros. Sci..

[18] Yokota H., Kudoh T., Suzuki T. (2003). Oxidation resistance of boronized MoSi_2_. Surf. Coat. Technol..

[19] Lange A., Braun R. (2014). Magnetron-sputtered oxidation protection coatings for Mo–Si–B alloys. Corros. Sci..

[20] Majumdar S., Dönges B., Gorr B., Christ H.-J., Schliephake D., Heilmaier M. (2015). Mechanisms of oxide scale formation on yttrium-alloyed Mo–Si–B containing fine-grained microstructure. Corros. Sci..

[21] Tian X., Guo X., Sun Z., Yin Z., Wang L. (2014). Formation of B-modified MoSi_2_ coating on pure Mo prepared through HAPC process. Int. J. Refract. Met. Hard Mater..

[22] Fu Q.-G., Li H., Wang Y.-J., Li K., Shi X.-H. (2009). B_2_O_3_ modified SiC–MoSi_2_ oxidation resistant coating for carbon/carbon composites by a two-step pack cementation. Corros. Sci..

[23] Pang J., Wang W., Zhou C. (2016). Microstructure evolution and oxidation behavior of B modified MoSi_2_ coating on Nb–Si based alloys. Corros. Sci..

[24] Wang C., Li K., Huo C., He Q., Shi X. (2018). Oxidation behavior and microstructural evolution of plasma sprayed La_2_O_3_-MoSi_2_-SiC coating on carbon/carbon composites. Surf. Coat. Technol..

[25] Sun L., Fu Q., Fang X.-Q., Sun J. (2018). A MoSi_2_-based composite coating by supersonic atmospheric plasma spraying to protect Nb alloy against oxidation at 1500 °C. Surf. Coat. Technol..

[26] Yanagihara K., Maruyama T., Nagata K. (1996). Effect of third elements on the pesting suppression of Mo-Si-X intermetallics (X = Al, Ta, Ti, Zr and Y). Intermetalics.

[27] Wang C., Li K., He Q., He D., Huo C., Su Y., Shi X., Li H. (2019). Oxidation and ablation protection of plasma sprayed LaB_6_-MoSi_2_-ZrB_2_ coating for carbon/carbon composites. Corros. Sci..

[28] Wang L., Fu Q., Liu N., Shan Y. (2016). Supersonic plasma sprayed MoSi_2_-ZrB_2_ antioxidation coating for SiC–C/C composites. Surf. Eng..

[29] Mao J., Ding S., Li Y., Li S., Liu F., Zeng X., Cheng X.-D. (2019). Preparation and investigation of MoSi_2_/SiC coating with high infrared emissivity at high temperature. Surf. Coat. Technol..

[30] Yan J., Liu L., Mao Z., Xu H., Wang Y. (2014). Effect of Spraying Powders Size on the Microstructure, Bonding Strength, and Microhardness of MoSi2 Coating Prepared by Air Plasma Spraying. J. Therm. Spray Technol..

[31] Sun J., Li T., Zhang G.-P. (2019). Effect of thermodynamically metastable components on mechanical and oxidation properties of the thermal-sprayed MoSi2 based composite coating. Corros. Sci..

[32] Zhang G., Sun J., Fu Q. (2020). Effect of mullite on the microstructure and oxidation behavior of thermal-sprayed MoSi_2_ coating at 1500 °C. Ceram. Int..

[33] Wu H., Li H., Lei Q., Fu Q.-G., Ma C., Yao D.-J., Wang Y.-J., Sun C., Wei J.-F., Han Z.-H. (2011). Effect of spraying power on microstructure and bonding strength of MoSi_2_-based coatings prepared by supersonic plasma spraying. Appl. Surf. Sci..

[34] Jia Y., Li H., Fu Q., Zhao Z., Sun J. (2017). Ablation resistance of supersonic-atmosphere-plasma-spraying ZrC coating doped with ZrO_2_ for SiC-coated carbon/carbon composites. Corros. Sci..

[35] Veytizou C. (2001). Zircon formation from amorphous silica and tetragonal zirconia: Kinetic study and modelling. Solid State Ionics.

[36] Chu Y., Li H.-J., Luo H., Li L., Qi L. (2015). Oxidation protection of carbon/carbon composites by a novel SiC nanoribbon-reinforced SiC–Si ceramic coating. Corros. Sci..

[37] Fu Q.-G., Jing J.-Y., Tan B.-Y., Yuan R.-M., Zhuang L., Li L. (2016). Nanowire-toughened transition layer to improve the oxidation resistance of SiC–MoSi_2_–ZrB_2_ coating for C/C composites. Corros. Sci..

[38] Lee W.Y., More K.L., Lara-Curzio E. (2005). Multilayered Oxide Interphase Concept for Ceramic-Matrix Composites. J. Am. Ceram. Soc..

[39] Sun C., Li H., Luo H., Xie J., Zhang J., Fu Q. (2013). Effect of Y_2_O_3_ on the oxidation resistant of ZrSiO_4_/SiC coating prepared by supersonic plasma spraying technique for carbon/carbon composites. Surf. Coat. Technol..

[40] Chu Y., Fu Q., Li H., Li K., Zou X., Gu C. (2011). Influence of SiC nanowires on the properties of SiC coating for C/C composites between room temperature and 1500 °C. Corros. Sci..

